# A reduction in sedentary behaviour in obese women during pregnancy reduces neonatal adiposity: the DALI randomised controlled trial

**DOI:** 10.1007/s00125-019-4842-0

**Published:** 2019-03-06

**Authors:** Mireille N. M. van Poppel, David Simmons, Roland Devlieger, F. Andre van Assche, Goele Jans, Sander Galjaard, Rosa Corcoy, Juan M. Adelantado, Fidelma Dunne, Jürgen Harreiter, Alexandra Kautzky-Willer, Peter Damm, Elisabeth R. Mathiesen, Dorte M. Jensen, Lise-Lotte Andersen, Mette Tanvig, Annunziata Lapolla, Maria G. Dalfra, Alessandra Bertolotto, Ewa Wender-Ozegowska, Agnieszka Zawiejska, David Hill, Frank J. Snoek, Judith G. M. Jelsma, Gernot Desoye

**Affiliations:** 1 Department of Public and Occupational Health, Amsterdam Public Health Research Institute, Amsterdam UMC, Vrije Universiteit Amsterdam, Amsterdam, the Netherlands; 2 Institute of Sport Science, University of Graz, Mozartgasse 14, 8010 Graz, Austria; 3 Macarthur Clinical School, Western Sydney University, Sydney, NSW Australia; 4 Institute of Metabolic Science, Addenbrooke’s Hospital, Cambridge, UK; 5 Department of Development and Regeneration: Pregnancy, Fetus and Neonate, KU Leuven, Leuven, Belgium; 6 Department of Gynaecology and Obstetrics, University Hospitals Leuven, Leuven, Belgium; 7 Division of Obstetrics and Prenatal Medicine, Department of Obstetrics and Gynaecology, Erasmus University Medical Center, Rotterdam, the Netherlands; 8 Institut de Recerca de l’Hospital de la Santa Creu i Sant Pau, Barcelona, Spain; 9 Centro de Investigación Biomédica en Red (CIBER) Bioengineering, Biomaterials and Nanotechnology, Instituto de Salud Carlos III (ISCIII), Madrid, Spain; 10 Galway Diabetes Research Centre, National University of Ireland, Galway, Ireland; 11 College of Medicine, Nursing and Health Sciences, National University of Ireland, Galway, Ireland; 12 Gender Medicine Unit, Endocrinology and Metabolism, Department of Internal Medicine III, Medical University of Vienna, Vienna, Austria; 13 Center for Pregnant Women with Diabetes, Departments of Endocrinology and Obstetrics, Rigshospitalet, Institute of Clinical Medicine, Faculty of Health and Medical Sciences, University of Copenhagen, Copenhagen, Denmark; 14 Steno Diabetes Center Odense, Odense University Hospital, Odense, Denmark; 15 Department of Gynaecology and Obstetrics, Odense University Hospital, Odense, Denmark; 16 Department of Clinical Research, Faculty of Health Sciences, University of Southern Denmark, Odense, Denmark; 17 Region of Southern Denmark, Denmark; 18 Dipartimento di Medicina, Università Degli Studi di Padova, Padua, Italy; 19 Department of Clinical and Experimental Medicine, Azienda Ospedaliero Universitaria Pisana, Pisa, Italy; 20 Medical Faculty I, Poznań University of Medical Sciences, Poznań, Poland; 21 Recherche en Santé Lawson SA, Bronschhofen, Switzerland; 22 Department of Medical Psychology, Amsterdam Public Health Research Institute, Amsterdam UMC, Vrije Universiteit Amsterdam, Amsterdam, the Netherlands; 23 Department of Obstetrics and Gynaecology, Medical University of Graz, Graz, Austria

**Keywords:** Lifestyle intervention, Mediation, Neonatal adiposity, Randomised controlled trial

## Abstract

**Aims/hypothesis:**

Offspring of obese women are at increased risk of features of the metabolic syndrome, including obesity and diabetes. Lifestyle intervention in pregnancy might reduce adverse effects of maternal obesity on neonatal adiposity.

**Methods:**

In the Vitamin D And Lifestyle Intervention for Gestational Diabetes Mellitus (GDM) Prevention (DALI) lifestyle trial, 436 women with a BMI ≥29 kg/m^2^ were randomly assigned to counselling on healthy eating (HE), physical activity (PA) or HE&PA, or to usual care (UC). In secondary analyses of the lifestyle trial, intervention effects on neonatal outcomes (head, abdominal, arm and leg circumferences and skinfold thicknesses, estimated fat mass, fat percentage, fat-free mass and cord blood leptin) were assessed using multilevel regression analyses. Mediation of intervention effects by lifestyle and gestational weight gain was assessed.

**Results:**

Outcomes were available from 334 neonates. A reduction in sum of skinfolds (−1.8 mm; 95% CI −3.5, −0.2; *p* = 0.03), fat mass (−63 g; 95% CI −124, −2; *p* = 0.04), fat percentage (−1.2%; 95% CI −2.4%, −0.04%; *p* = 0.04) and leptin (−3.80 μg/l; 95% CI −7.15, −0.45; *p* = 0.03) was found in the HE&PA group, and reduced leptin in female neonates in the PA group (−5.79 μg/l; 95% CI −11.43, −0.14; *p* = 0.05) compared with UC. Reduced sedentary time, but not gestational weight gain, mediated intervention effects on leptin in both the HE&PA and PA groups.

**Conclusions/interpretation:**

The HE&PA intervention resulted in reduced adiposity in neonates. Reduced sedentary time seemed to drive the intervention effect on cord blood leptin. Implications for future adiposity and diabetes risk of the offspring need to be elucidated.

**Trial registration:**

ISRCTN70595832.

**Electronic supplementary material:**

The online version of this article (10.1007/s00125-019-4842-0) contains peer-reviewed but unedited supplementary material, which is available to authorised users.

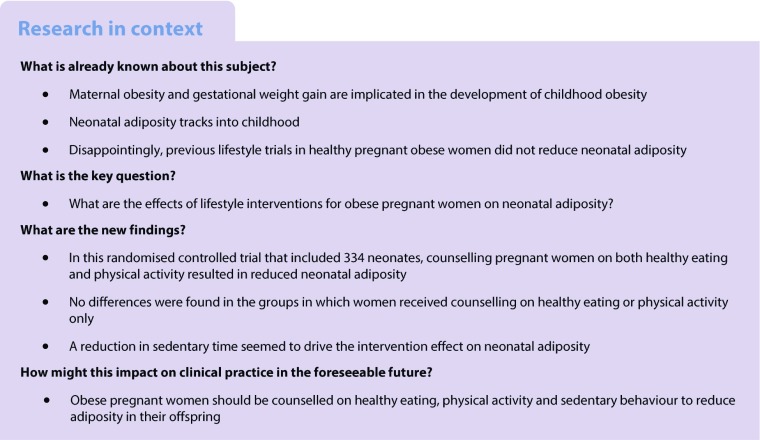



## Introduction

Globally, an estimated 41 million children under 5 years of age were either overweight or obese in 2014, which is a major public health concern [1].

Obesity during the lifetime, including childhood, tracks back to the early postnatal period [2] and, as recently reported, even back to birth: the Hyperglycemia and Adverse Pregnancy Outcome (HAPO) follow-up study, including 4832 children at a mean age of 11.4 years, demonstrated the association of adiposity at birth with adiposity in late childhood [3]. Furthermore, a recent study showed that being large for gestational age at birth is related to an increased risk of adolescent obesity [4]. Therefore, reducing adiposity at birth is a key target for the prevention of obesity later in life.

Maternal obesity and excessive gestational weight gain in pregnancy are implicated in the development of neonatal obesity [5–8]. Lifestyle intervention in pregnant, obese women might reduce the adverse effects of maternal obesity on offspring adiposity, possibly associated with a reduction in gestational weight gain. Two previous studies have shown that treatment of women with mild gestational diabetes results in a reduction in neonatal adiposity [9, 10], although it was not maintained into childhood [11, 12]. Disappointingly, lifestyle trials in healthy pregnant obese women did not find such effects on neonatal adiposity [13–15], possibly owing to insufficient change in lifestyle behaviours or minimal changes in gestational weight gain [16, 17].

In the pan-European Vitamin D And Lifestyle Intervention for Gestational Diabetes Mellitus (GDM) Prevention (DALI) trial, motivational interviewing (MI)-based counselling on healthy eating (HE), physical activity (PA) and a combination of the two (HE&PA) was compared with usual care (UC) [18–20]. Primary outcomes were gestational weight gain, fasting glucose and insulin sensitivity. We previously reported improvements in lifestyle behaviour in all three intervention groups, in addition to a substantial reduction in gestational weight gain in the HE&PA group, but no changes in fasting glucose or insulin sensitivity [20].

Here we tested whether the lifestyle interventions altered neonatal anthropometry and cord blood leptin, as a marker of adiposity [21], both secondary outcomes of the DALI lifestyle trial. We also investigated whether changes in neonatal adiposity were primarily mediated through changes in lifestyle, gestational weight gain, or both. The findings would allow targeted counselling of pregnant women to improve the long-term weight of their offspring and, hence, address a major public health concern.

## Methods

### Design and participants

**Fig. 1:**
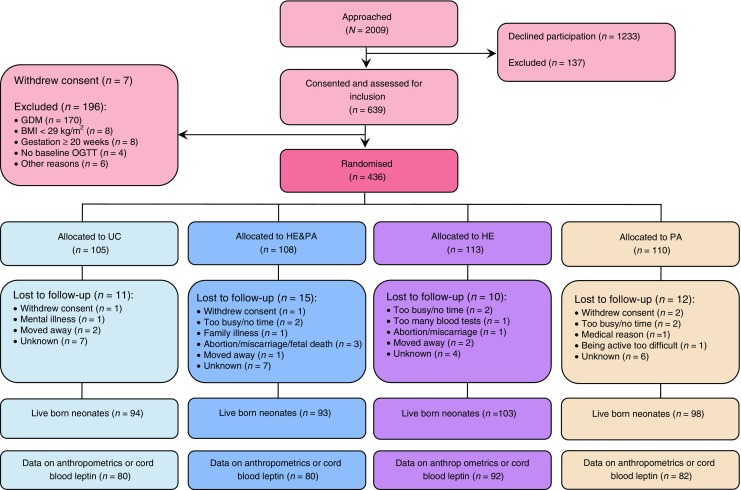
Eligibility, randomisation and follow-up of women and neonates in the DALI lifestyle trial. GDM, gestational diabetes mellitus

Originally, the DALI study was designed as a trial with a 2 × (2 × 2) factorial design, including a vitamin D trial and a lifestyle trial [18]. The two trials were not conducted in parallel as originally planned but, because of logistical reasons, the vitamin D trial started after the lifestyle trial had finished. The current paper describes secondary analyses of the DALI lifestyle trial, a multicentre parallel randomised trial conducted in nine European countries (Austria, Belgium, Denmark [Odense, Copenhagen], Ireland, Italy [Padua, Pisa], the Netherlands, Poland, Spain and the UK) during 2012–2015. The study was prospectively registered as an RCT on 21 November 2011 (ISRCTN70595832). Local ethics committee approval and written informed consent of all women was obtained. Pregnant women with a pre-pregnancy BMI ≥29 kg/m^2^, <20 weeks of gestation, a singleton pregnancy and age ≥ 18 years were invited to participate. Exclusions included diagnosis with early gestational diabetes [22], pre-existing diabetes and chronic medical conditions. The numbers of women excluded and included are shown in the flow chart (Fig. 1).

### Randomisation, masking and interventions

Women were randomised to HE&PA, HE, PA or UC, using a computerised random number generator, pre-stratified for site. Staff involved with measurements, but not participants, were blinded to the intervention.

In the intervention groups, participants were assigned to a single coach, with whom they discussed five physical activity and/or seven healthy eating messages, depending on group allocation, and were advised to keep gestational weight gain below 5 kg [18]. Most lifestyle coaches had a background in behavioural change, healthy eating and/or physical activity. Prior to the start of the study, a 2 day central training course in Cambridge (UK) was offered to the coaches of all sites, which was led by experienced MI trainers. This was repeated a few months later to review the coaches’ MI competency, share experiences and receive feedback on a role play. The DALI coaches received in total 32 h of MI training. Their MI skills in the trial were evaluated by external MI experts [23]. MI-guided coaching of the trial participants took place during five face-to-face sessions of 30–45 min each, alternated with up to four optional telephone calls. In the UC group, participants received no DALI interventions.

### Outcomes

#### Neonatal anthropometry

Neonatal weight and length were measured at birth and, within 48 h, head, abdomen, upper- and lower-arm and upper- and lower-leg circumferences were measured. Research nurses received central training for the measurements at the beginning of the trial and participated in a standardisation meeting within 6 months after trial start. Skinfold thickness was measured at four sites, i.e. triceps, subscapular, suprailiac and quadriceps, with a Harpenden skinfold calliper [18], and values summed. Each skinfold measurement was repeated once and if a difference of more than 0.2 mm was registered a third measurement was performed and the average of the three was taken. Time between birth and measurements was registered (in h). Estimated fat mass (in g) was calculated using a validated equation for neonates [24], with all neonates classified as ‘non-Hispanic’. Estimated fat-free mass (in g) was calculated as total body mass minus estimated fat mass. Estimated fat percentage was calculated by dividing estimated fat mass by total body mass × 100.

#### Cord blood leptin concentration

Venous cord blood samples were taken immediately after delivery. Locally, blood samples were stored at −20°C or colder until transported frozen to the central trial laboratory in Graz, Austria. There, they were stored at −70°C for 3 years maximum (the first women included) and 2 months minimum (last women included). Leptin concentrations were quantified by solid-phase sandwich ELISA (E05–086-96; EIASON, Graz, Austria), according to the manufacturer’s instructions. Analytical sensitivity was 1.0 ng/ml; intra- and inter-assay coefficients of variation (low/high concentrations) were 6.0%/6.9% and 11.6%/8.7%, respectively.

#### Maternal height, weight and lifestyle characteristics

Maternal height was determined at baseline with a stadiometer (SECA 206; SECA, Birmingham, UK). Women were weighed on calibrated electronic scales (SECA 888 and 877) at baseline and at 24–28 weeks and 35–37 weeks of gestation. BMI was calculated as weight (in kg) divided by the square of height (in m). Gestational weight gain was defined as the change in objectively measured weight from baseline to 24–28 weeks and from baseline to 35–37 weeks.

At baseline and at 24–28 and 35–37 weeks, maternal physical activity was assessed with the validated Pregnancy Physical Activity Questionnaire [25], which assesses the time spent sedentary (watching TV or video, sitting and reading, talking or on the phone) and in light, moderate or vigorous physical activity measured in metabolic equivalent of task (MET) h/week. Nutrition was assessed using a bespoke, short food-frequency questionnaire covering key foods, linked to the intervention messages [26].

#### Covariates

Information on possible covariates was collected in the baseline questionnaire or from medical files: parity (nulliparous vs multiparous), education (low vs high), smoking status (yes vs no), pre-pregnancy BMI, mode of delivery (Caesarean vs vaginal delivery) and gestational age at birth.

### Patient involvement

Representatives of the target group were interviewed in the developmental stage of the trial about their preferences of intervention content, modality, frequency and location [27]. Patients were not involved in the actual conduct of the study, but study participants provided feedback on the burden of the intervention and their experiences with the study in general, as part of a process evaluation [23]. Patient organisations are actively involved in the dissemination of the results to the lay public.

### Statistical analyses

Data presented in this paper are secondary outcomes of the DALI lifestyle trial [18]. All neonatal outcomes presented in this paper were pre-specified in the protocol, with the exception of estimated fat mass, fat-free mass and fat percentage, which were calculated based on pre-specified variables.

Neonatal loss to follow-up was defined as no data on both neonatal anthropometry and cord blood leptin. Differences between the study sample and participants with loss to follow-up and differences between the intervention groups and the UC group were tested using Student’s *t* test for continuous variables or χ^2^ tests for categorical variables.

To test for intervention effects, multilevel analyses were undertaken with a two level structure: individual and site. A modified intention-to-treat approach was used, without any imputation of missing data. A sensitivity analysis was conducted with multiple imputation under missing-at-random assumptions. Analyses of neonatal anthropometric variables were always adjusted for the time after birth (in h). Additional adjustment of analyses by parity, pre-pregnancy BMI, education, smoking, sex, gestational age at birth and mode of delivery did not change the results and are therefore not shown. Effect dependency by sex was assessed for all outcomes. An interaction with *p* < 0.10 was considered relevant and, when present, analyses were performed for both sexes separately. All analyses were performed with IBM SPSS, version 20 (IBM, Armonk, NY, USA), with a two-sided *p* value below 0.05 considered significant. No adjustments for multiple comparisons were made.

Post hoc power calculations showed that a difference of 2.0 mm (SD 5.0) in sum of skinfolds could be found with an α value of 5% and power of 80% with 98 women per group, and a difference of 1.5% (SD 3.5) in estimated fat percentage could be found with 85 women in each group.

Mediation by lifestyle factors was assessed in multiple parallel mediation models [28, 29]. This allows the determination of the lifestyle factor with the strongest indirect effect [28]. The model was limited to the six lifestyle factors that changed significantly due to the interventions (moderate-to-vigorous physical activity, sedentary behaviour, sugared drink consumption, vegetable consumption, carbohydrate intake and portion size) [20]. Subsequently, mediation by gestational weight gain was assessed using a simple mediation model. Sex dependency of all pathways in the mediation models was tested. Models with the sum of skinfolds or estimated fat percentage as outcome were adjusted for the time of measurement (in h) after birth. Models with gestational weight gain as mediator were adjusted for the number of days between measurements.

## Results

Figure 1 shows the randomisation and follow-up of participants in the trial. A total of 436 women were included in the trial. Participants lost to follow-up (*n* = 102) were not different from those for whom neonatal data were available (*n* = 334) (Table 1). Maternal characteristics were comparable between intervention groups (Table 1), except that gestational weight gain was lower in the HE&PA group compared with the UC group.

**Table 1:** Maternal and neonatal characteristics per intervention group

Characteristic	UC	HE&PA	HE	PA	Neonatal LTFU
Maternal^a^					
Age, years	31.9 ± 5.6	32.5 ± 5.3	32.4 ± 5.5	31.7 ± 4.9	31.5 ± 5.5
Multiparous	35 (44)	42 (53)	51 (55)	38 (46)	47 (46)
European descent	71 (89)	69 (86)	79 (86)	68 (83)	91 (89)
Living with partner	76 (95)	74 (93)	90 (98)	77 (94)	93 (91)
Higher education	45 (56)	43 (54)	55 (60)	46 (56)	50 (49)
Maternal smoking	13 (16)	7 (9)	17 (19)	13 (16)	17 (17)
Paternal smoking	24 (30)	22 (28)	37 (40)	27 (33)	33 (33)
Pre-pregnancy BMI, kg/m^2^	33.7 ± 3.7	33.6 ± 3.6	34.2 ± 4.6	33.8 ± 3.9	33.4 ± 3.9
Gestational weight gain at 35–37 weeks, kg	8.6 ± 4.6	6.4 ± 3.9^b^	7.6 ± 4.9	8.2 ± 4.9	8.3 ± 4.4
Gestational diabetes at 24–28 weeks	8 (10)	11 (14)	13 (14)	9 (11)	12 (16)
Insulin or metformin after 24–28 weeks	1 (1)	3 (4)	1 (1)	0 (0)	3 (3)
Neonatal^c^					
Female sex	45 (56)	41 (51)	41 (45)	39 (48)	33 (52)
Gestational age at birth, weeks	39.7 ± 1.4	39.8 ± 1.4	39.6 ± 1.7	39.6 ± 1.4	38.9 ± 4.2
Birthweight, g	3581 ± 510	3480 ± 504	3485 ± 629	3469 ± 512	3399 ± 546
Birthweight <2500 g	4 (5)	1 (1)	5 (6)	2 (3)	4 (7)
Birthweight >4000 g	18 (23)	15 (19)	18 (20)	13 (16)	7 (11)

Data are *n* (%) or means ± SD

^a^Maternal groups: UC, *n*=80; HE&PA, *n*=80; HE, *n*=92; PA, *n*=82; neonatal LTFU, *n*=102 (*n*=100 for paternal smoking; *n*=73 for gestational diabetes)

^b^Gestational weight gain in the HE&PA group was the only characteristic different from the UC group (*p*=0.002)

^c^Neonatal groups: UC, *n*=80; HE&PA, *n*=80; HE, *n*=91; PA, *n*=81; neonatal LTFU, *n*=64 (*n*=62 for birthweight <2500 g and >4000 g)

LTFU, loss to follow-up

### Effects of interventions on neonatal anthropometry and cord blood leptin

Neonatal anthropometry and cord blood leptin per intervention group are described in Table 2. The HE&PA intervention was associated with smaller thigh (−0.7 mm, *p* = 0.04) and flank (−0.5 mm, *p* = 0.03) skinfold thicknesses, sum of skinfolds (−1.8 mm, *p* = 0.03), as well as a reduced estimated fat mass (−63 g, *p* = 0.04) and estimated fat percentage (−1.2%, *p* = 0.04) but no effect on estimated fat-free mass, head or abdominal circumferences compared with UC (Table 3). Furthermore, cord blood leptin levels were reduced in this intervention group (−3.80 μg/l, *p* = 0.03). In the HE group, no significant differences were observed compared with UC. In the PA group, no differences in neonatal anthropometry were observed. However, a small but statistically significant reduction in cord blood leptin was found in female neonates only (−5.79 μg/l, *p* = 0.05). Results from the sensitivity analysis, using complete data, showed similar intervention effects on neonatal anthropometry (electronic supplementary material [ESM] Table 1).

**Table 2:** Neonatal anthropometry and cord blood leptin according to intervention group

Variable	UC		HE&PA		HE		PA	
	*n*	Mean±SD	*n*	Mean±SD	*n*	Mean±SD	*n*	Mean±SD
Anthropometry								
Triceps skinfold, mm	69	5.3 ± 1.3	70	5.0 ± 1.3	74	5.3 ± 1.5	69	5.3 ± 1.6
Subscapular skinfold, mm	71	5.0 ± 1.4	70	4.6 ± 1.1	74	5.0 ± 1.4	68	4.9 ± 1.4
Thigh skinfold, mm	70	6.9 ± 1.9	69	6.2 ± 1.7	75	6.6 ± 2.1	68	6.5 ± 2.1
Flank skinfold, mm	70	4.5 ± 1.7	69	3.9 ± 1.1	75	4.4 ± 1.6	68	4.3 ± 1.3
Sum of skinfolds, mm	69	21.6 ± 5.2	69	19.8 ± 4.3	73	21.2 ± 5.8	68	21.0 ± 5.4
Head circumference, cm	78	34.8 ± 1.7	79	34.8 ± 1.5	90	34.6 ± 2.0	80	34.8 ± 1.9
Abdominal circumference, cm^a^	73	33.4 ± 2.6	70	33.0 ± 2.7	76	33.3 ± 3.2	74	32.9 ± 2.7
Male	31	33.6 ± 2.7	33	32.7 ± 2.9	43	33.4 ± 3.5	37	33.2 ± 2.5
Female	42	33.3 ± 2.6	37	33.3 ± 2.5	33	33.2 ± 2.8	36	32.6 ± 3.0
Upper-arm circumference, cm	73	11.4 ± 1.2	71	11.2 ± 1.0	77	11.5 ± 1.3	71	11.5 ± 1.3
Lower-arm circumference, cm	73	10.0 ± 1.0	70	9.9 ± 1.0	77	10.0 ± 1.0	71	9.9 ± 1.0
Thigh circumference, cm	73	15.4 ± 1.7	71	15.0 ± 2.1	77	15.4 ± 1.8	71	15.3 ± 1.7
Calf circumference, cm	73	11.6 ± 1.2	70	11.5 ± 1.2	77	11.6 ± 1.2	71	11.6 ± 1.1
Estimated fat mass, g	67	511 ± 181	68	451 ± 171	69	492 ± 213	65	463 ± 190
Estimated fat percentage, %	67	13.8 ± 3.5	68	12.6 ± 3.4	69	13.2 ± 4.18	65	12.9 ± 3.8
Estimated fat-free mass, g	67	3111 ± 339	68	3029 ± 351	69	3105 ± 410	65	3021 ± 3547
Cord blood								
Leptin, μg/l^a^	64	12.19 ± 13.01	50	8.29 ± 6.33	70	10.00 ± 7.95	57	9.10 ± 6.88
Male	31	7.57 ± 4.94	25	6.80 ± 4.93	37	8.21 ± 4.80	29	8.31 ± 7.37
Female	33	16.52 ± 16.52	25	9.77 ± 7.27	33	12.02 ± 10.12	28	9.91 ± 6.36

Differences between groups are shown in Table 3

^a^Data on male and female neonates are provided separately for variables for which an interaction of intervention effect with sex was found (*p*<0.10)

**Table 3:** Differences in neonatal outcomes per intervention group compared with UC group

Neonatal outcome	HE&PA vs UC		HE vs UC		PA vs UC	
	β (95% CI)	*p* value	β (95% CI)	*p* value	β (95% CI)	*p* value
Anthropometry^a^						
Triceps skinfold, mm	−0.3 (−0.8, 0.1)	0.14	−0.2 (−0.6, 0.3)	0.49	−0.1 (−0.5, 0.4)	0.74
Subscapular skinfold, mm	−0.4 (−0.8, 0.1)	0.10	−0.1 (−0.5, 0.3)	0.65	−0.2 (−0.7, 0.2)	0.28
Thigh skinfold, mm	−0.7 (−1.3, −0.03)	0.04	−0.4 (−1.1, 0.2)	0.17	−0.5 (−1.2, 0.1)	0.11
Flank skinfold, mm	−0.5 (−0.9, −0.1)	0.03	−0.2 (−0.6, 0.2)	0.38	−0.2 (−0.6, 0.3)	0.49
Sum of skinfolds, mm	−1.8 (−3.5, −0.2)	0.03	−0.8 (−2.5, 0.8)	0.33	−1.0 (−2.6, 0.7)	0.25
Head circumference, cm	0.0 (−0.5, 0.6)	0.99	−0.1 (−0.6, 0.5)	0.78	0.0 (−0.5, 0.6)	0.93
Abdominal circumference, cm	−0.3 (−1.3, 0.5)	0.45	−0.2 (−1.0, 0.7)	0.72	−0.8 (−1.7, 0.1)	0.08
Male	−1.3 (−2.7, 0.02)^†^	0.05	−0.3 (−1.6, 1.0)	0.63	−1.0 (−2.3, 0.3)	0.14
Female	0.3 (−0.9, 1.5)^†^	0.64	0.1 (−1.2, 1.3)	0.89	−0.9 (−2.1, 0.4)	0.17
Upper-arm circumference, cm	−0.2 (−0.6, 0.2)	0.31	0.1 (−0.3, 0.4)	0.79	0.0 (−0.4, 0.4)	0.87
Lower-arm circumference, cm	−0.2 (−0.5, 0.1)	0.26	0.0 (−0.3, 0.3)	1.00	−0.2 (−0.5, 0.2)	0.38
Thigh circumference, cm	−0.5 (−1.0, 0.1)	0.13	−0.1 (−0.7, 0.5)	0.76	−0.2 (−0.8, 0.4)	0.44
Calf circumference, cm	−0.1 (−0.5, 0.2)	0.49	−0.0 (−0.4, 0.4)	0.98	−0.1 (−0.5, 0.3)	0.63
Estimated fat mass, g	−63 (−124, −2)	0.04	−23 (−84, 38)	0.45	−58 (−119, 4)	0.07
Estimated fat percentage, %	−1.2 (−2.4, −0.04)	0.04	−0.7 (−1.8, 0.5)	0.27	−1.0 (−2.2, 0.2)	0.09
Estimated fat-free mass, g	−78 (−198, 42)	0.20	−2 (−122, 118)	0.98	−82 (−203, 40)	0.19
Cord blood						
Leptin, μg/l	−3.80 (−7.15, −0.45)	0.03	−2.01 (−5.09, 1.07)	0.20	−2.90 (−6.15, 0.34)	0.08
Male	−0.77 (−3.74, 2.19)	0.61	0.64 (−2.05, 3.33)	0.64	0.74 (−2.11, 3.59)^†^	0.61
Female	−6.50 (−12.31, −0.69)	0.03	−3.83 (−9.21, 1.55)	0.16	−5.79 (−11.43, −0.14)^†^	0.05

*p* values for the intervention effect are based on multilevel regression models, with the individual and site as levels

^a^Analyses with neonatal anthropometry outcomes were adjusted for time (in h) after birth of the measurement. Further adjusting of analyses by parity, pre-pregnancy BMI, education, smoking, neonatal sex, gestational age at birth and mode of delivery did not change the results

^†^*p*<0.10 for interaction with neonatal sex

### Mediation effects of lifestyle and gestational weight gain on neonatal anthropometry and cord blood leptin

In contrast to the multilevel analyses for intervention effects, mediation analyses did not detect significant interaction with neonatal sex (data not shown); therefore, sexes were combined in all further mediation analyses.

**Fig. 2:**
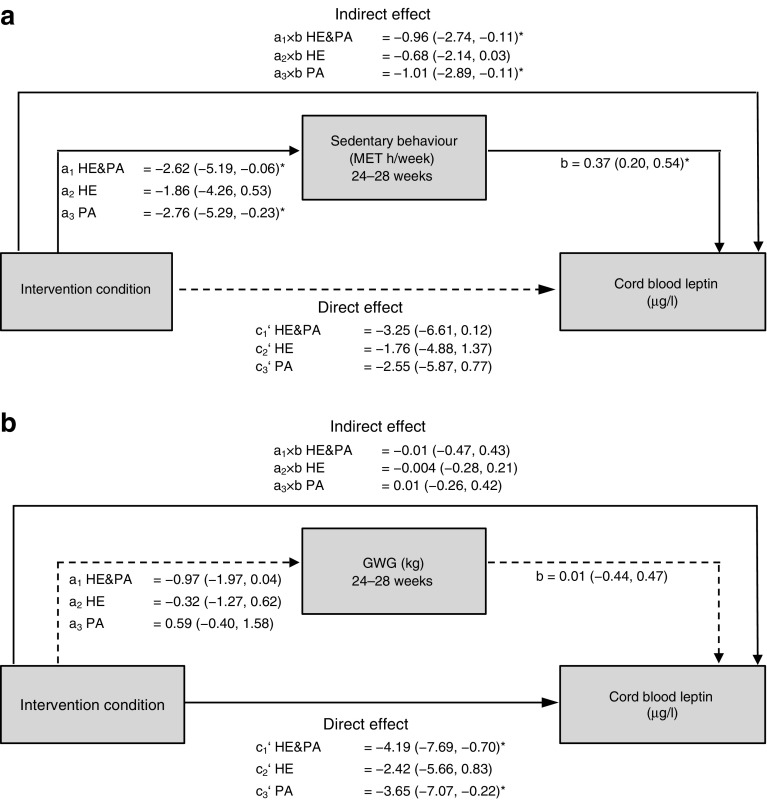
Schematic diagram of the results of simple mediation by sedentary behaviour at 24–28 weeks (*n*=239) (**a**) and gestational weight gain (GWG) at 24–28 weeks (*n*=238) (**b**) on cord blood leptin. The path coefficients between intervention condition, mediators (sedentary behaviour in **a** and GWG in **b**) and cord blood leptin are shown. Statistically significant path coefficients (*p*<0.05) are indicated with asterisks and with solid lines. Non-significant paths are indicated with dashed lines. Indirect effects are calculated as the product of the coefficients of the (**a**) and (**b**) paths (a×b). MET, metabolic equivalent of task

First, we examined whether changes in lifestyle mediated the intervention effects in the HE&PA and/or PA group on sum of skinfolds, estimated fat percentage and leptin (ESM Tables 2–4). In parallel mediation models, including all six lifestyle factors, no mediation was found for sum of skinfolds or estimated fat percentage (ESM Tables 2, 3). Sedentary behaviour was the only lifestyle factor mediating intervention effects on leptin (ESM Table 4). Mediation of the intervention effect on leptin by sedentary behaviour was further analysed in a simple mediation model. From baseline to 24–28 weeks, sedentary behaviour was reduced in both the HE&PA and PA interventions (Fig. 2a), and cord blood leptin was increased among women who had more sedentary behaviour (β = 0.37; 95% CI 0.20, 0.54). The reduction in sedentary behaviour mediated the effects of the HE&PA and PA interventions on leptin (indirect effect HE&PA −0.96; 95% CI −2.74, −0.11; indirect effect PA −1.01; 95% CI −2.89, −0.11); consequently, the interventions were no longer directly associated with leptin in an independent manner (Fig. 2a). Changes in sedentary behaviour from baseline to 35–37 weeks did not mediate intervention effects on leptin (ESM Table 4).

Second, we tested whether gestational weight gain mediated intervention effects on the sum of skinfolds, estimated fat percentage and cord blood leptin (ESM Table 5). No mediation by gestational weight gain at 24–28 or at 35–37 weeks was found. Gestational weight gain at 24–28 weeks was not significantly associated with leptin (β = 0.01; 95% CI −0.44, 0.47) (Fig. 2b). The reductions in gestational weight gain at 24–28 weeks did not mediate the effects of the HE&PA or PA interventions on leptin (indirect effect HE&PA −0.01; 95% CI −0.47, 0.43; indirect effect PA 0.01; 95% CI −0.26, 0.42), and the direct associations of the interventions with leptin remained significant (Fig. 2b). Sedentary behaviour at 24–28 weeks was not associated with gestational weight gain at 24–28 (β = −0.02; 95% CI −0.06, 0.03) or at 35–37 weeks (β = 0.01; 95% CI −0.06, 0.08).

## Discussion

Associations between maternal lifestyle and neonatal adiposity exist [30–32], but causality has not been demonstrated. Here we used an RCT design to demonstrate the consequences of changing lifestyle in pregnancy on neonatal adiposity. The combined HE&PA intervention resulted in a reduction of thigh, flank skinfold thickness, sum of skinfolds, estimated fat mass, fat percentage and cord blood leptin. There was no effect on lean mass. In the PA intervention, a reduction in leptin was found in female neonates. Effects on leptin in both intervention groups were mediated through a reduction in sedentary behaviour. Changes in gestational weight gain alone did not mediate these intervention effects.

The DALI lifestyle trial is the first comprehensive study to demonstrate beneficial changes in neonatal anthropometric measures, indicating a reduction in neonatal adiposity, a finding validated by a reduction in cord blood leptin. No reduction in fat-free mass was found, which was previously suggested as a possible risk factor for future cardiovascular and metabolic disease [33, 34]. Previous large RCTs of combined HE&PA interventions reported positive changes in physical activity and diet [16, 17] but not in neonatal body composition [13, 14] or cord blood leptin [14]. A low glycaemic diet intervention resulted in a lower thigh circumference but did not affect any other neonatal body composition outcome [35]. None of these previous interventions focused on reducing sedentary behaviour, whereas DALI HE&PA and PA interventions highlighted the importance of reducing sitting time during counselling sessions [18].

The clinical relevance of the reduction in neonatal adiposity for future child health is unclear. Longitudinal studies demonstrated an association between lower neonatal adiposity and subsequent lower adiposity in infancy [36] and childhood [3, 37, 38]. The long-term follow-up of the offspring of the DALI lifestyle trial will help assess the relevance of our intervention effects on the neonate for future obesity and health.

The mediation analyses suggest that gestational weight gain does not mediate intervention effects on neonatal outcomes. This finding differs from previous longitudinal cohort studies that reported associations of gestational weight gain with neonatal adiposity [8, 39, 40]. This may be due, in part, to residual confounding in observational studies that was excluded by our RCT design. Furthermore, it might be that only early pregnancy gestational weight gain—obviously not influenced by our interventions—is related to neonatal adiposity [39], although such associations in later pregnancy have been reported [8]. The most likely explanation, however, is that our study included only obese women, and excessive gestational weight gain has no or a much smaller effect on neonatal fat mass in this group [41, 42].

The metabolic pathway(s) linking changes in sedentary behaviour and neonatal adiposity remain(s) to be identified. Maternal glucose associates with risk of large-for-gestational-age neonates [43], but the DALI HE&PA and PA interventions had no effect on fasting and post-load glucose levels [20]. However, a reduction in sedentary time was associated with reduced cord blood leptin levels. In previous observational studies, more sedentary time was associated with increased maternal leptin [44], lipid levels [44, 45] and C-reactive protein levels [45] but not with insulin resistance or glucose [44, 46]. Reduced inflammation might be involved in the pathway between sedentary behaviour and offspring adiposity, a hypothesis worth testing.

Some strengths and limitations of our study need considering. A strength of the pan-European DALI lifestyle trial is its unique design in assessing effects of counselling interventions on healthy eating and physical activity separately or in combination. Although data were missing for some neonates, women providing data on neonatal anthropometry or cord blood leptin were similar to the total study sample. Furthermore, intervention effects were confirmed in sensitivity analysis using complete data. Our results are therefore applicable to white, obese (BMI ≥29 kg/m^2^) pregnant women throughout Europe. A further strength is the use of leptin as a biomarker of neonatal adiposity. Although also produced by the placenta, cord blood leptin levels correlate with neonatal adiposity (Spearman’s correlation coefficient *r* = 0.46 with estimated fat mass in this study [data not shown]) [21]. Leptin levels reflect total fetal fat mass and not only subcutaneous fat, in contrast to skinfold thickness measurements. However, more direct measures of neonatal adiposity, such as air displacement plethysmography, would have been preferred. The use of self-reported data on lifestyle factors in the mediation analyses could be considered a limitation of our study. Therefore, it is possible that the counselling intervention a woman received influenced her answers to questions on lifestyle. We did not predefine a main outcome for the analyses presented in this paper, which may be regarded as a limitation. A further limitation is the limited power of the study. Despite this, we found significant intervention effects on several outcomes that are highly related to each other, which adds confidence to the findings observed. However, it does have consequences for the sample size of a future study, which should be adequately powered in order to be able to replicate/validate our findings.

Intervention effects on neonatal adiposity were due to reduced sedentary behaviour but not increased moderate-to-vigorous physical activity. This might imply that increasing time spent in light-intensity physical activity is important and is likely easier for obese women to implement in their daily lives. Importantly, current guidelines for physical activity in pregnancy focus recommendations on maintaining sufficient levels of moderate-to-vigorous physical activity [47, 48]. Should our present findings be confirmed for other populations across the BMI and ethnicity ranges, guidelines on physical activity in pregnancy will need modification to include a reduction in sedentary behaviour.

Since many recent lifestyle interventions in pregnancy, including the DALI lifestyle trial, have failed to show beneficial effects on maternal glucose metabolism and incidence of gestational diabetes, it has been suggested that future efforts might instead be best invested in pre-conception lifestyle interventions. However, our results show that whilst gestational diabetes risk was not reduced, lifestyle intervention in pregnancy is relevant for reducing neonatal adiposity and may have implications for future childhood obesity. The lack of intervention effects on birthweight also emphasises that birthweight is a crude measure of neonatal adiposity and demonstrates the importance of measuring body composition instead.

In summary, the combined HE&PA intervention resulted in less subcutaneous fat in neonates and lower cord blood leptin levels. These effects were not mediated by gestational weight gain, despite a substantial reduction of 2.0 kg in the HE&PA group [20]. However, reduced sedentary behaviour mediated the intervention effect on leptin. The mechanisms linking sedentary behaviour and neonatal adiposity, and the implications for future child obesity, need to be elucidated.

## Electronic supplementary material

Tables (PDF 474 kb)

## Data Availability

Data are available from the corresponding author on reasonable request.

## Abbreviations

DALI: Vitamin D And Lifestyle Intervention for Gestational Diabetes Mellitus (GDM) Prevention
HE: Counselling on healthy eating (intervention group)
HE&PA: Counselling on healthy eating and physical activity (intervention group)
MI: Motivational interviewing
PA: Counselling on physical activity (intervention group)
UC: Usual care (intervention group)
