# Supplementary material for: A reduction in sedentary behaviour in obese women during pregnancy reduces neonatal adiposity: the DALI randomised controlled trial
Source: Diabetologia. 2019 Mar 6;62(6):915–25. doi: 10.1007/s00125-019-4842-0 (PMC6509072; doi:10.1007/s00125-019-4842-0)
Supplement: Supplementary file 1 — (PDF 474 kb) [file 125_2019_4842_MOESM1_ESM.pdf]

Supplementary Material

Supplement to: van Poppel et al. “**A reduction in sedentary behavior in obese women reduces neonatal adiposity: The DALI randomized controlled trial**”

Table of contents

Supplementary Table 1.	Differences in neonatal outcomes per intervention group compared to usual care group, using complete data generated with multiple imputation.	2
Supplementary Table 2.	Multiple parallel mediation model with sum of skin folds as outcome.	3
Supplementary Table 3.	Multiple parallel mediation model with estimated fat percentage as outcome.	4
Supplementary Table 4.	Multiple parallel mediation model with cord blood leptin as outcome.	5
Supplementary Table 5.	Simple mediation model of gestational weight gain as mediator and sum of skin folds, estimated fat percentage and cord blood leptin as outcomes.	6

.

Supplementary Table 1. Differences in neonatal outcomes per intervention group compared to usual care group, using complete data generated with multiple imputation.

	HE&PA vs UC		HE vs UC		PA vs UC	
Anthropometry†	**Beta (95% CI)**	**P value***	**Beta (95% CI)**	**P value***	**Beta (95% CI)**	**P value***
Triceps skinfold — mm	-0.3 (-0.7; 0.1)	0.13	-0.1 (-0.5; 0.3)	0.69	0.04 (-0.4; 0.5)	0.84
Subscapular skinfold — mm	-0.3 (-0.7; 0.1)	0.16	-0.01 (-0.4; 0.4)	0.96	-0.01 (-0.4; 0.4)	0.98
Thigh skinfold — mm	**-0.4 (-0.8; -0.03)**	**0.04**	-0.1 (-0.5; 0.3)	0.63	-0.03 (-0.5; 0.5)	0.92
Flank skinfold — mm	-0.4 (-1.0; 0.2)	0.18	-0.2 (-0.8; 0.4)	0.48	-0.03 (-0.7; 0.6)	0.92
Sum of skinfolds — mm	**-1.4 (-2.8; -0.02)**	**0.047**	-0.4 (-1.9; 1.0)	0.57	-0.03 (-1.5; 1.4)	0.97
Head circumference — cm	0.1 (-0.4; 0.6)	0.69	-0.2 (-0.8; 0.3)	0.43	0.1 (-0.4; 0.6)	0.68
Abdominal circumference — cm	-0.2 (-0.9; 0.6)	0.62	-0.3 (-1.0; 0.4)	0.44	-0.5 (-1.3; 0.3)	0.21
Upper arm circumference — cm	-0.1 (-0.5; 0.3)	0.57	-0.05 (-0.4; 0.3)	0.80	0.2 (-0.2; 0.5)	0.37
Lower arm circumference — cm	-0.1 (-0.4; 0.2)	0.41	-0.1 (-0.4; 0.2)	0.60	-0.1 (-0.4; 0.3)	0.67
Thigh circumference — cm	-0.3 (-0.8; 0.2)	0.30	-0.3 (-0.8; 0.3)	0.32	0.04 (-0.5; 0.6)	0.90
Calf circumference — cm	-0.04 (-0.4; 0.3)	0.83	-0.1 (-0.4; 0.3)	0.68	0.1 (-0.3; 0.4)	0.67
Estimated fat mass — g	-32 (-94; 31)	0.33	-25 (-92; 41)	0.46	-12 (-77; 53)	0.72
Estimated fat percentage — %	**-1.2 (-2.4; -0.05)**	**0.04**	-0.7 (-1.8; 0.5)	0.27	-1.0 (-2.4; 0.2)	0.09
Estimated fat free mass — g	-78 (-197; 42)	0.20	-2 (-121; 118)	0.98	-82 (-203; 40)	0.19
Cord blood						
Leptin — µg/l	**-5.45 (-8.80; -2.11)**	**0.001**	**-4.52 (-7.59; -1.46)**	**0.004**	**-4.83 (-8.59; -1.06)**	**0.01**

UC denotes usual care, HE healthy eating, and PA physical activity.

Multiple imputations were performed in SPSS (version 22), using 40 imputations with a maximum of 50 iterations. Predictive mean matching was used as the model for scale variables.

* P values for the intervention effect are based on the pooled results of the multiple imputed datasets. Datasets were analyzed using multilevel regression models, with the individual and site as levels.

† Analyses with neonatal anthropometry outcomes were adjusted for time in hours after birth of the measurement.

Supplementary Table 2. Multiple parallel mediation model with sum of skin folds as outcome.

	Intervention effect on the mediator (a-path)			Effect of mediator on the outcome (b-path)	Indirect effect (path a*b)			Direct intervention effect on the outcome (c'-path)		
	Estimate (95% CI)			Estimate (95% CI)	Estimate (95% CI)			Estimate (95% CI)		
Mediator variable at 24-28 weeks of gestation (N=223)	HE&PA	HE	PA		HE&PA	HE	PA	HE&PA	HE	PA
MVPA, MET-h/wk	10.87 (-7.06; 28.79)	8.77 (-9.56; 27.10)	10.39 (-8.18; 28.96)	0.007 (-0.005; 0.02)	-0.04 (-0.35; 0.08)	-0.03 (-0.33; 0.07)	-0.04 (-0.38; 0.06)			
Sedentary behavior, MET-h/wk	**-2.52 (-4.95; -0.09)**	-1.67 (-4.16; 0.81)	-1.03 (-3.56; 1.49)	-0.05 (-0.15; 0.05)	0.03 (-0.24; 0.35)	0.02 (-0.14; 0.29)	0.01 (-0.11; 0.28)			
Sugary drinks, portions /wk	0.52 (-2.66; 3.70)	-3.05 (-6.31; 0.20)	1.88 (-1.41; 5.18)	-0.04 (-0.17; 0.09)	-0.02 (-0.60; 0.20)	0.13 (-0.23; 0.65)	-0.08 (-0.59; 0.10)	**-2.23 (-4.07; -0.39)**	-1.42 (-3.33; 0.47)	-0.58 (-2.48; 1.33)
Vegetables, portions /wk	2.48 (-0.95; 5.91)	3.03 (-0.48; 6.53)	-0.23 (-3.79 3.32)	0.003 (-0.07; 0.08)	0.01 (-0.16; 0.27)	0.01 (-0.20; 0.36)	-0.001 (-0.13; 0.08)			
Carbohydrates, portions /wk	-1.56 (-8.53; 5.41)	**-7.70 (-14.82; -0.57)**	-4.79 (-12.01; 2.43)	0.02 (-0.03; 0.07)	-0.02 (-0.55; 0.16)	-0.12 (-0.70; 0.24)	-0.08 (-0.58; 0.12)			
Portion size, portions /wk	-1.73 (-6.55; 3.09)	**-6.32 (-11.25; -1.39)**	-0.92 (-5.92; 4.08)	-0.005 (-0.10; 0.09)	0.01 (-0.32; 0.53)	0.03 (-0.61; 0.87)	0.004 (-0.23; 0.37)			
Mediator variable at 35-37 weeks of gestation (N=205)	HE&PA	HE	PA		HE&PA	HE	PA	HE&PA	HE	PA
MVPA, MET-h/wk	**19.36 (2.26; 36.46)**	7.83 (-9.44; 25.10)	11.05 (-6.53; 28.63)	-0.01 (-0.02; 0.01)	-0.11 (-0.62; 0.10)	-0.04 (-0.37; 0.06)	-0.06 (-0.46; 0.07)			
Sedentary behavior, MET-h/wk	**-3.90 (-6.91; -0.88)**	-0.26 (-3.31; 2.78)	-2.14 (-5.24; 0.96)	-0.01 (-0.11; 0.08)	0.05 (-0.31; 0.50)	0.003 (-0.14; 0.24)	0.03 (-0.18; 0.37)			
Sugary drinks, portions /wk	**-4.42 (-7.54; -1.30)**	**-3.71 (-6.85; -0.56)**	-0.23 (-3.43; 2.98)	0.06 (-0.07; 0.19)	-0.26 (-1.12; 0.24)	-0.22 (-1.11; 0.18)	-0.01 (-0.54; 0.21)	**-2.34 (-4.37; -0.30)**	-1.09 (-3.12; 0.94)	-0.83 (-2.84; 1.19)
Vegetables, portions /wk	1.00 (-2.60; 4.60)	**4.32 (0.69; 7.95)**	-0.76 (-4.46; 2.94)	-0.01 (-0.09; 0.06)	-0.01 (-0.26; 0.05)	-0.06 (-0.44; 0.15)	0.01 (-0.06; 0.22)			
Carbohydrates, portions /wk	**-8.74 (-15.09; -2.40)**	**-10.13 (-16.53; -3.72)**	**-7.55 (-14.07; -1.03)**	0.01 (-0.05; 0.08)	-0.12 (-0.80; 0.49)	-0.13 (-0.93; 0.53)	-0.10 (-0.76; 0.39)			
Portion size, portions /wk	**-6.76 (-11.49; -2.03)**	**-7.65 (-12.42; -2.87)**	-3.72 (-8.58; 1.15)	-0.08 (-0.17; 0021)	0.51 (-0.03; 1.77)	0.58 (-0.03; 1.68)	0.28 (-0.04; 1.19)			

All analyses were adjusted for age at measurement in hours after birth.

Supplementary Table 3. Multiple parallel mediation model with estimated fat percentage as outcome.

	Intervention effect on the mediator (a-path)			Effect of mediator on the outcome (b-path)	Indirect effect (path a*b)			Direct intervention effect on the outcome (c'-path)		
	Estimate (95% CI)			Estimate (95% CI)	Estimate (95% CI)			Estimate (95% CI)		
Mediator variable at 24-28 weeks of gestation (N=223)	HE&PA	HE	PA		HE&PA	HE	PA	HE&PA	HE	PA
MVPA, MET-h/wk	10.87<br>(-7.06; 28.79)	8.77<br>(-9.56; 27.10)	10.39<br>(-8.18; 28.96)	-0.003<br>(-0.01; 0.01)	-0.03<br>(-0.28; 0.05)	-0.02<br>(-0.26; 0.05)	-0.03<br>(-0.30; 0.04)			
Sedentary behavior, MET-h/wk	**-2.52**<br>**(-4.95; -0.09)**	-1.67<br>(-4.16; 0.81)	-1.03<br>(-3.55; 1.49)	-0.01<br>(-0.08; 0.07)	0.02<br>(-0.19; 0.25)	0.01<br>(-0.11; 0.21)	0.01<br>(-0.08; 0.19)			
Sugary drinks, portions /wk	0.52<br>(-2.66; 3.70)	-3.05<br>(-6.31; 0.20)	1.88<br>(-1.41; 5.18)	-0.01<br>(-0.10; 0.08)	-0.01<br>(-0.36; 0.13)	0.04<br>(-0.27; 0.40)	-0.02<br>(-0.33; 0.15)	**-1.66**<br>**(-3.01;-0.32)**	-1.18<br>(-2.57; 0.21)	-0.91<br>(-2.31; 0.48)
Vegetables, portions /wk	2.48<br>(-0.95; 5.91)	3.03<br>(-0.48; 6.53)	-0.23<br>(-3.79; 3.32)	-0.01<br>(-0.06; 0.05)	-0.01<br>(-0.21; 0.12)	-0.02<br>(-0.21; 0.17)	0.001<br>(-0.08; 0.09)			
Carbohydrates, portions /wk	-1.56<br>(-8.53; 5.41)	**-7.70**<br>**(-14.82; -0.57)**	-4.79<br>(-12.01; 2.43)	0.002<br>(-0.03; 0.04)	-0.004<br>(-0.25; 0.16)	-0.02<br>(-0.36; 0.28)	-0.01<br>(-0.28; 0.17)			
Portion size, portions /wk	-1.73<br>(-6.55; 3.09)	**-6.32**<br>**(-11.25; -1.39)**	-0.92<br>(-5.92; 4.08)	0.01<br>(-0.06; 0.08)	-0.02<br>(-0.49; 0.17)	-0.09<br>(-0.69; 0.44)	-0.01<br>(-0.34; 0.13)			
Mediator variable at 35-37 weeks of gestation (N=205)	HE&PA	HE	PA		HE&PA	HE	PA	HE&PA	HE	PA
MVPA, MET-h/wk	**19.36**<br>**(2.26; 36.46)**	7.83<br>(-9.44; 25.10)	11.05<br>(-6.53; 28.63)	-0.002<br>(-0.01; 0.01)	-0.06<br>(-0.37; 0.09)	-0.02<br>(-0.22; 0.05)	-0.03<br>(-0.27; 0.06)			
Sedentary behavior, MET-h/wk	**-3.90**<br>**(-6.91; -0.88)**	-0.26<br>(-3.31; 2.78)	-2.14<br>(-5.24; 0.96)	-0.03<br>(-0.10; 0.03)	0.13<br>(-0.12; 0.60)	0.01<br>(-0.12; 0.28)	0.07<br>(-0.06; 0.44)			
Sugary drinks, portions /wk	**-4.42**<br>**(-7.54; -1.30)**	**-3.71**<br>**(-6.85; -0.56)**	-0.23<br>(-3.43; 2.98)	0.0001<br>(-0.09; 0.09)	-0.0002<br>(-0.52; 0.40)	-0.0002<br>(-0.47; 0.35)	0.000<br>(-0.18; 0.17)	**-1.98**<br>**(-3.40; -0.56)**	-0.69<br>(-2.11; 0.73)	-1.10<br>(-2.51; 0.31)
Vegetables, portions /wk	1.00<br>(-2.60; 4.60)	**4.32**<br>**(0.69; 7.95)**	-0.76<br>(-4.46; 2.94)	-0.02<br>(-0.08; 0.03)	-0.02<br>(-0.23; 0.04)	-0.11<br>(-0.42; 0.04)	0.02<br>(-0.05; 0.21)			
Carbohydrates, portions /wk	**-8.74**<br>**(-15.09; -2.40)**	**-10.13**<br>**(-16.53; -3.72)**	**-7.55**<br>**(-14.07; -1.03)**	0.01<br>(-0.03; 0.05)	-0.09<br>(-0.56; 0.27)	-0.10<br>(-0.64; 0.31)	-0.08<br>(-0.55; 0.23)			
Portion size, portions /wk	**-6.76**<br>**(-11.49; -2.03)**	**-7.65**<br>**(-12.43; -2.87)**	-3.72<br>(-8.58; 1.15)	-0.03<br>(-0.10; 0.04)	0.20<br>(-0.19; 0.99)	0.23<br>(-0.24; 0.94)	0.11<br>(-0.10; 0.67)			

All analyses were adjusted for age at measurement in hours after birth

Supplementary Table 4. Multiple parallel mediation model with cord blood leptin as outcome.

	Intervention effect on the mediator (a-path)			Effect of mediator on the outcome (b-path)	Indirect effect (path a*b)			Direct intervention effect on the outcome (c'-path)		
	Estimate (95% CI)			Estimate (95% CI)	Estimate (95% CI)			Estimate (95% CI)		
Mediator variable at 24-28 weeks of gestation (N=197)	HE&PA	HE	PA		HE&PA	HE	PA	HE&PA	HE	PA
MVPA, MET-h/wk	-0.90<br>(-19.28; 17.49)	4.92<br>(-12.33; 22.18)	16.78<br>(-0.98; 34.25)	0.02<br>(-0.01; 0.05)	-0.02<br>(-0.83; 0.41)	0.09<br>(-0.24; 1.19)	0.32<br>(-0.24; 1.57)			
Sedentary behavior, MET-h/wk	**-3.83**<br>**(-6.72; -0.94)**	-1.30<br>(-4.01; 1.41)	**-3.18**<br>**(-5.97; -0.39)**	**0.47**<br>**(0.26; 0.68)**	**-1.81**<br>**(-4.48; -0.47)**	-0.61<br>(-2.51; 0.44)	**-1.50**<br>**(-4.13; -0.18)**			
Sugary drinks, portions /wk	-1.68<br>(-3.80; 0.43)	**-3.30**<br>**(-5.29; -1.31)**	1.34<br>(-0.71; 3.38)	-0.24<br>(-0.60; 0.12)	0.41<br>(-0.10; 1.61)	0.80<br>(-0.22; 2.30)	-0.32<br>(-1.58; 0.08)	-1.37<br>(-5.64; 2.89)	-1.28<br>(-5.30; 2.74)	-2.79<br>(-6.88; 1.31)
Vegetables, portions /wk	3.56<br>(-0.34; 7.45)	**3.87**<br>**(0.21; 7.52)**	-0.21<br>(-3.97; 3.55)	-0.12<br>(-0.27; 0.04)	-0.41<br>(-1.22; 0.02)	**-0.45**<br>**(-1.47; -0.03)**	0.02<br>(-0.37; 0.52)			
Carbohydrates , portions /wk	**-7.04**<br>**(-13.48; -0.61)**	**-8.84**<br>**(-14.88; -2.80)**	-4.12<br>(-10.34; 2.10)	0.03<br>(-0.09; 0.16)	-0.23<br>(-1.33; 0.56)	-0.29<br>(-1.56; 0.68)	-0.14<br>(-1.19; 0.24)			
Portion size, portions /wk	**-5.54**<br>**(-9.90; -1.17)**	**-6.63**<br>**(-10.73; -2.53)**	-1.79<br>(-6.00; 2.43)	0.10<br>(-0.11; 0.31)	-0.54<br>(-2.05; 0.44)	-0.65<br>(-2.38; 0.58)	-0.18<br>(-1.24; 0.23)			
Mediator variable at 35-37 weeks of gestation (N=180)	HE&PA	HE	PA		HE&PA	HE	PA	HE&PA	HE	PA
MVPA, MET-h/wk	8.18<br>(-8.43; 24.78)	7.42<br>(-8.29; 23.14)	**17.88**<br>**(1.45; 34.31)**	0.003<br>(-0.04; 0.05)	0.02<br>(-0.34; 0.74)	0.02<br>(-0.31; 0.71)	0.05<br>(-0.64; 1.19)			
Sedentary behavior, MET-h/wk	**-3.62**<br>**(-6.93; -0.32)**	-1.57<br>(-4.69; 1.56)	-2.26<br>(-5.53; 1.01)	0.12<br>(-0.10; 0.34)	-0.43<br>(-1.89; 0.21)	-0.19<br>(-1.35; 0.11)	-0.27<br>(-1.62; 0.16)			
Sugary drinks, portions /wk	**-3.87**<br>**(-6.90; -0.83)**	**-3.87**<br>**(-6.74; -1.00)**	-1.12<br>(-4.12; 1.88)	0.13<br>(-0.24; 0.50)	-0.50<br>(-3.04; 0.86)	-0.50<br>(-3.31; 0.80)	-0.15<br>(-2.39; 0.31)	--3.28<br>(-7.99; 1.43)	-0.68<br>(-5.28; 3.92)	-4.52<br>(-9.19; 0.15)
Vegetables, portions /wk	2.01<br>(-1.73; 5.74)	2.89<br>(-0.65; 6.42)	1.54<br>(-2.15; 5.23)	-0.07<br>(-0.26; 0.12)	-0.14<br>(-1.13; 0.13)	-0.20<br>(-1.34; 0.14)	-0.11<br>(-1.04; 0.08)			
Carbohydrates, portions /wk	**-6.53**<br>**(-12.75; -0.32)**	**-13.33**<br>**(-19.21; -7.45)**	**-9.24**<br>**(-15.39; -3.09)**	-0.03<br>(-0.18; 0.13)	0.18<br>(-0.42; 1.70)	0.37<br>(-1.11; 2.46)	0.26<br>(-0.64; 2.02)			
Portion size, portions /wk	**-4.62**<br>**(-9.13; -0.12)**	**-8.55**<br>**(-12.82; -4.29)**	-3.70<br>(-8.16; 0.76)	0.03<br>(-0.24; 0.29)	-0.12<br>(-1.51; 0.77)	-0.22<br>(-2.01; 1.60)	-0.10<br>(-1.25; 0.60)			

Supplementary Table 5. Simple mediation model of gestational weight gain as mediator and sum of skin folds, estimated fat percentage and cord blood leptin as outcomes.

	Intervention effect on the mediator (a-path) Estimate (95% CI)			Effect of mediator on the outcome (b-path) Estimate (95% CI)	Indirect effect (path a*b) Estimate (95% CI)			Direct intervention effect on the outcome (c'-path) Estimate (95% CI)		
	HE&PA	HE	PA		HE&PA	HE	PA	HE&PA	HE	PA
Sum of skin folds										
GWG 24-28 wks	**-1.31**	-0.62	0.10	-0.002	0.002	0.001	-0.0002	**-1.86**	-1.09	-1.11
N=265	**(-2.23; -0.38)**	(-1.56; 0.33)	(-0.84; 1.04)	(-0.22; 0.22)	(-0.31; 0.37)	(-0.18; 0.22)	(-0.13; 0.14)	**(-3.54; -0.19)**	(-2.77; 0.60)	(-2.79; 0.57)
GWG 35-37 wks	**-2.65**	-0.85	-0.41	0.04	-0.10	-0.03	-0.02	**-1.96**	-0.68	-0.80
N=252	**(-4.25; -1.04)**	(-2.49; 0.78)	(-2.05; 1.23)	(-0.09; 0.17)	(-0.50; 0.28)	(-0.34; 0.08)	(-0.28; 0.08)	**(-3.67; -0.26)**	(-2.38; 1.02)	(-2.51; 0.90)
Estimated fat percentage										
GWG 24-28 wks	**-1.31**	-0.62	0.10	0.06	-0.07	-0.03	0.01	-1.18	-0.79	-1.11
N=265	**(-2.23; -0.38)**	(-1.56; 0.33)	(-0.84; 1.04)	(-0.10; 0.22)	(-0.34; 0.14)	(-0.26; 0.05)	(-0.07; 0.16)	(-2.38; 0.03)	(-2.00; 0.43)	(-2.32; 0.10)
GWG 35-37 wks	**-2.65**	-0.85	-0.41	0.06	-0.15	-0.05	-0.02	**-1.21**	-0.37	-0.90
N=252	**(-4.25; -1.04)**	(-2.49; 0.78)	(-2.05; 1.23)	(-0.03; 0.15)	(-0.48; 0.09)	(-0.30; 0.04)	(-0.25; 0.07)	**(-2.39; -0.02)**	(-1.55; 0.81)	(-2.08; 0.29)
Cord blood leptin										
GWG 24-28 wks	-0.97	-0.32	0.59	0.01	-0.01	-0.004	0.01	**-4.19**	-2.42	**-3.65**
N=238	(-1.97; 0.04)	(-1.27; 0.62)	(-0.40; 1.58)	(-0.44; 0.47)	(-0.47; 0.43)	(-0.28; 0.21)	(-0.26; 0.42)	**(-7.69; -0.70)**	(-5.66; 0.83)	**(-7.07; -0.22)**
GWG 35-37 wks	-1.53	-0.16	0.45	0.04	-0.06	-0.01	0.02	**-3.61**	-1.73	**-3.77**
N=224	(-3.30; 0.23)	(-1.82; 1.51)	(-1.34; 2.23)	(-0.23; 0.31)	(-0.59; 0.30)	(-0.32; 0.17)	(-0.13; 0.43)	**(-7.12; -0.11)**	(-5.01; 1.56)	**(-7.28; -0.25)**

All analyses were adjusted for the number of days between weight measurements. Analyses with sum of skin folds and estimated fat percentage as outcomes were adjusted for age at measurement in hours after birth in addition.
